# Gall-Stones with Hepatic Abscess

**Published:** 1893-05

**Authors:** Sydney A. Dunham

**Affiliations:** 72 West Chippewa Street; Buffalo, N. Y.


### GALL-STONES WITH HEPATIC ABSCESS.

BY SYDNEY A. DUNHAM, M. D., Buffalo, N. Y.

The fact that these hepatic affections so frequently go unrecognized, until an autopsy is necessary to reveal the real state of affairs, or to confirm a doubtful diagnosis, gives me a reason for presenting this subject for discussion. From reading, observation, and experience upon this subject, I am convinced that the liver, which is the largest and most vascular gland in the body, with more functions than any other, is worthy of our closest attention and study.

My interest in this organ was first excited, when a student, by attending an autopsy held by half a dozen of the older physicians over a man who had been treated for several months by the same number of doctors, for a variety of diseases dependent upon obscure symptoms; and the causes of death would have been as numerous only for a post mortem, which revealed an abscess of the liver, after the intestinal tract had been carefully scrutinized for suspected disease.

Mrs. W., aged fifty-five, widow, sent for me February 6, 1892; she had had digestive disturbances for about one year, food distressed her, bowels constipated, and had lost in flesh.

She gave a history of having had one attack of hepatic colic a year and a half previous, but now the uneasiness was confined to umbilical region with no hepatic tenderness. On February 9th, I was sent for and found the patient with fever following a chill, and the next day a broncho-pneumonia of right lung was evident; as resolution in the right lung took place, the left lung became involved, and the patient becoming alarmed, the family physician, Dr. H. Z. Zelrington, of Susquehanna, was sent for. He said the patient had never been treated for liver affection, as she had had no symptoms of hepatic disease.

On the twenty-first day of her illness, the lungs cleared up and the patient was improving; on the second day following, she was jaundiced; on the third day, jaundice increased with clay-colored stools; on fourth day, purpura hemorrhagia, covering entire body except the face, appeared; fifth day, patient the same but weaker; sixth day, suddenly she became comatose, and died the next day.

Post-mortem revealed ruptured gall-bladder, with gall-stones and hepatic abscess. There were no liver disturbances which were noticed or complained of while the lung trouble was present; but when jaundice occurred, the patient wished to lay upon her right side, and said there was a heavy feeling there.

Without an autopsy, the Board of Vital Statistics, or anybody else, would have had any idea of the cause of death.

One day, while at the Buffalo Medical and Surgical Dispensary, I was called out to examine a woman on Broadway. She was large and fleshy, and was suffering from pain in the right hypochondriac region. After an examination and history of the case, I pronounced the trouble neuralgia, the patient a neurasthenic, and told her I could help her.

Later, I was told that the patient was no better, that pain came and went just the same as it had for a long time, and that Dr. Hartwig had pronounced the trouble gall-stones, and wished to remove them, but the patient was not ready for such an operation. She went to the country, and, after a month, the physician there was obliged to open an abscess on the right side, which continued to discharge pus until Dr. Park operated upon her, one year ago last December, at the General Hospital, removing several gall-stones from the gall-bladder. The biliary fistula, which still remains, has been a physiological study for me during the past year. If I did not consider the bile an important agent in intestinal digestion, I would have thought, at times, that the entire amount secreted was discharged from the side, as it was impossible to find dressings sufficient to absorb the amount poured out in one night. At my request, the patient would put on clean dressings before each meal, and four hours afterwards the dressings would hardly be stained with bile, but during the intervals of digestion, when the current of bile is directed to the gall-bladder, there was bile in abundance coming from the fistulous opening.

The discharge has rapidly lessened in quantity and changed in quality the past two months, and at one time it stopped altogether for a week, but was reopened by vigorous exercise; since then it has been intermittent. The third day after it stopped flowing from the side, the patient was taken with a diarrhea, which was painless, yet the stools were profuse, thin, without odor, and of natural color, lasting three days.

This attack could only be accounted for either by the passage of a biliary calculus, which allowed a free flow of bile into the intestines, or the opening of some new channel into the digestive tract. The purging, I believe, must have been due to the purgative action of the unusual large amount of bile entering the alimentary canal. Since then the bowels have moved regularly without the aid of laxatives, which were before resorted to. At the present time the fistulous discharge is very slight, and we are anxiously waiting for it to disappear entirely. I might make use of a few interesting reports upon this subject.

Dr. Poulsen, in his report of 9,172 autopsies, says that only nine per cent. of the individuals examined, in which gall-stones were found, had exhibited signs of their existence during lifetime.

According to Naunyn, twenty-five per cent. of all women over sixty years of age have gall-stones.

Bollinger found gall-stones in 5.4 per cent. of cadavers; the proportion of women to men was 5.2 per cent.

W. A. Edwards, in the Sacramento *Medical Times*, reported a case of a man dying of pneumonia; the autopsy revealed eighty-eight gall-stones weighing 490 grains. There were no liver symptoms, except fulness in region of gall-bladder.

We are naturally led from the foregoing to the following conclusion: The fact that symptoms are so often wanting in gall-bladder disease, is easily explained when we look for the nerve supply which is so poor that the organ is almost insensible to pain. The ordinary symptoms which we look for and depend upon for a diagnosis seldom appear, but an autopsy reveals the fatal biliary accumulation. While gall-stones remain in the gall-bladder and cystic duct, they give us little inconvenience, and they, as well as hepatic abscess, may go from beginning to end without hepatic disturbances. In order that our death certificates represent what they should to the Board of Health, we should be more like the Frenchman in diagnosis, when it is uncertain during life, settle it afterward by a post-mortem examination.

72 West Chippewa Street.

